# A case of drug‐induced Stevens‐Johnson syndrome‐like eruption predominating in mucosa: A case report

**DOI:** 10.1002/ccr3.2806

**Published:** 2020-06-27

**Authors:** Akihiro Ishiguro, Tomoyuki Shibata, Takeshi Yanagishita, Hiroyuki Takama, Rimi Uchida, Yuichiro Ohshima, Daisuke Watanabe

**Affiliations:** ^1^ Department of Dermatology Aichi Medical University Nagakute Japan

**Keywords:** atypical SJS, drug eruption, meloxicam, mycoplasma, Stevens‐Johnson syndrome, tizanidine

## Abstract

Atypical Stevens‐Johnson syndrome (SJS) is a variant of SJS with complete absence of or only few cutaneous manifestations. It usually affects children with Mycoplasma‐induced respiratory infection. It was unique because our case was induced by drug and occurred in an adult.

## Graphical abstract

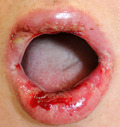

## 1 CASE REPORT

A 58‐year‐old woman developed cheilitis and blisters on vulva. Drug‐induced lymphocyte stimulation test (DLST) results were positive for tizanidine and meloxicam. She was diagnosed with atypical Stevens‐Johnson syndrome (SJS). Atypical SJS is a variant of SJS with complete absence of or only few cutaneous manifestations.

Atypical SJS, also called Fuchs syndrome, usually occurs after *Mycoplasma* infection. Atypical SJS (a rare variant of SJS), presents with exclusive mucosal involvement with complete absence of or only few cutaneous manifestations.^1^ We report a case of drug‐induced atypical SJS.

**Figure 1:**
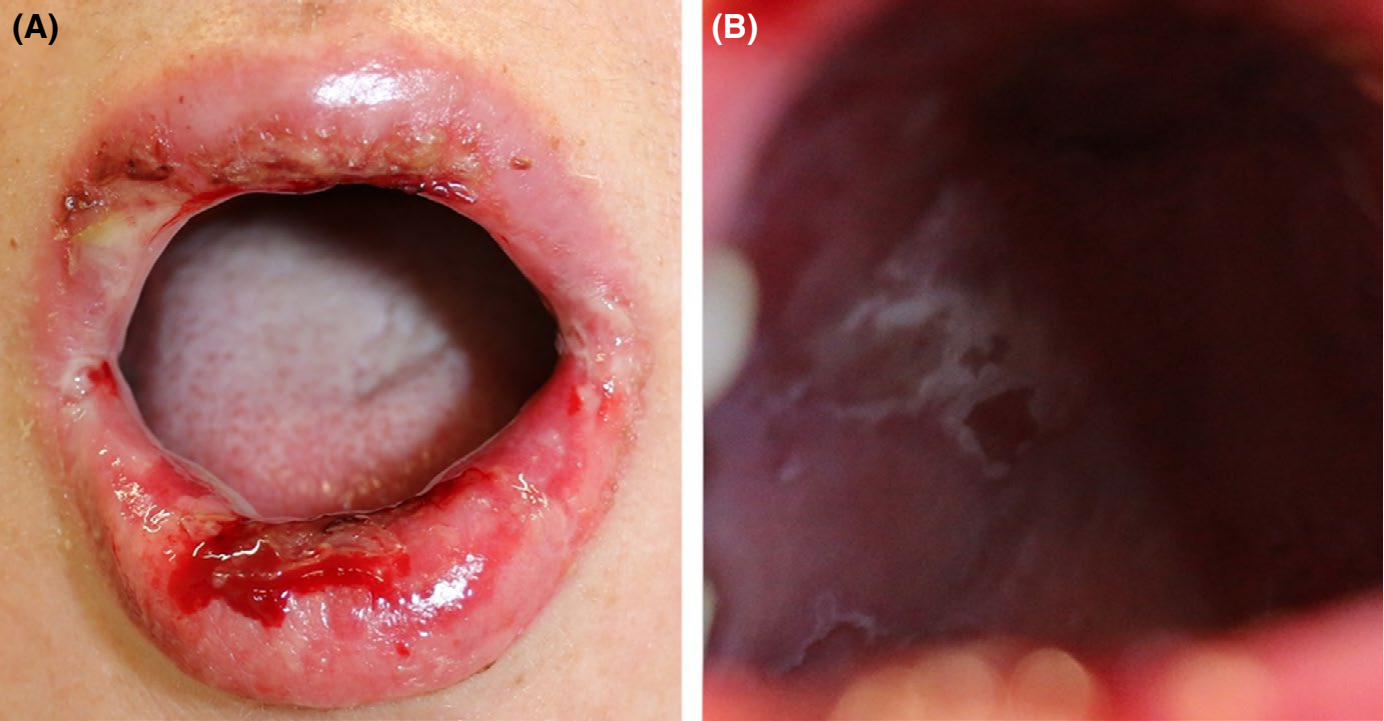
Clinical features of the patient showing (A) erosions and crusts on the lips and (B) furred upper plate of mouth

**Figure 2:**
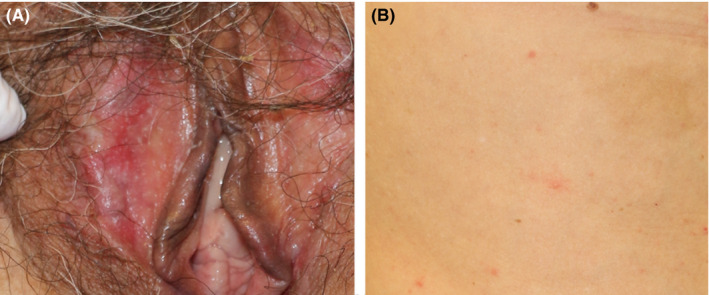
Clinical features of the patient showing (A) vulvar erosions and (B) mildly reddish papule on the right side of the abdomen

**Figure 3:**
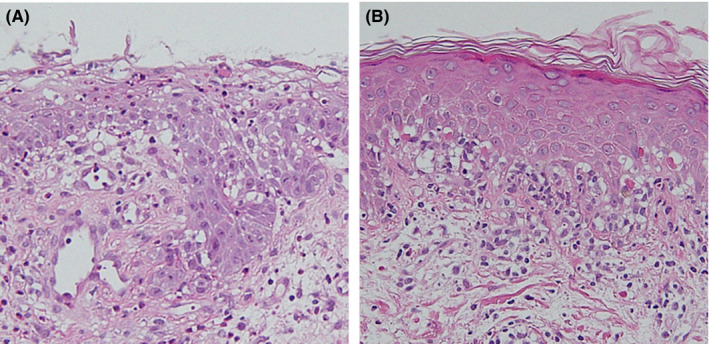
Histopathological examination showing skin biopsy specimens from (A) the lips and (B) the abdomen. Interface changes are observed in the basal layers of the epidermis along with epidermal necrosis

A 58‐year‐old woman presented with a rash on her trunk and extremities. She reported a history of meloxicam and tizanidine use following dental treatment prior to the appearance of the rash. Two days thereafter, she developed stomatitis and lip blisters. She was diagnosed with suspected herpes labialis at an Internal Medicine clinic and received oral valacyclovir with vidarabine ointment for 3 days; however, her symptoms persisted. Three days thereafter, the rash appeared on the trunk and extremities, and she was referred to our department. She revealed a history of uterine dysplasia but denied any allergies. She had no fever. Physical examination showed erosions, edema, and blisters on her lips and vulva (Figures 1 and 2A). Mildly reddish papules, without target lesion, without vesicles, were observed on the right side of her abdomen and thighs (Figure 2B). The white blood cell count was 8800 cells/µL, eosinophil count was 132/µL, and CRP level was 3.24 mg/dL. Biopsy specimens were obtained from the lip erosions and from the papules over her trunk. Histopathological examination of the lip specimens revealed an interface change at the base of the epidermis, epidermal necrosis (Figure 3A), and neutrophilic infiltration of blood vessel walls. Histopathological examination of abdominal specimens showed similar findings (Figure 3B). *Mycoplasma* antibodies at admission and 12 days after admission were negative. Herpes simplex virus antibody at admission and 12 days after admission was also negative. Drug‐induced lymphocyte stimulation test (DLST) results were positive for tizanidine (409%) and meloxicam (346%). Based on these findings, she was diagnosed with SJS and was admitted to our hospital. Prednisolone drip was initiated at a dose of 70 mg/d, and her symptoms gradually improved, and she was discharged 18 days after admission.

Stevens‐Johnson syndrome is a rare but serious and often life‐threatening drug‐induced skin disorder. Extensive erythema, erosions, and blisters occur throughout the body, including the mucous membranes of the lips, oral cavity, eyes, and vulva, along with high fever and malaise. SJS is commonly drug induced, although it may occasionally be secondary to infection.

Atypical SJS is a variant of SJS with complete absence of or only few cutaneous manifestations. It usually affects children with *Mycoplasma*‐induced respiratory infection.^2^ Ravin et al^3^ first reported three cases of atypical SJS. Unlike the typical presentation of SJS, these patients do not show severe symptoms and show a good response to treatment. All three cases reported in the aforementioned study occurred secondary to infections in children. In most cases, the infection was mycoplasma infection. Previous reports have described that all cases of atypical SJS improved with antibiotic treatment, and several patients could be discharged within 2 weeks.^3^, ^4^, ^5^ This report describes a case of atypical SJS in a patient who presented with mild cutaneous manifestations and was discharged without residual effects following successful systemic steroid therapy. Although this clinical presentation of our case resembled atypical SJS previously described, it was unique because our case was induced by drug and occurred in an adult.

## CONFLICT OF INTEREST

None declared.

## AUTHOR CONTRIBUTIONS

DW: involved in project administration. DW: involved in supervision. AI, TS, and DW: involved in validation. Akihiro Ishiguro, TS, and DW: wrote the original drafted manuscript. AI, TS, TY, HT, RU, YO, and DW: wrote, reviewed, and edited the manuscript. All authors approved the manuscript to be published and agreed to be accountable for all aspects of the work in ensuring that questions related to the accuracy or integrity of any part of the work are appropriately investigated and resolved.
